# The promise and challenge of personalized medicine: aging populations, complex diseases, and unmet medical need

**DOI:** 10.3325/cmj.2012.53.207

**Published:** 2012-06

**Authors:** Adriano M Henney

**Affiliations:** 1 Obsidian Biomedical Consulting Ltd., Macclesfield, UK; 2 Director of German Virtual Liver Network

## Abstract

**Abstract:**

The concept of personalized medicine is not new. It is being discussed with increasing interest in the medical, scientific, and general media because of the availability of advanced scientific and computational technologies, and the promise of the potential to improve the targeting and delivery of novel medicines. It is also being seen as one approach that may have a beneficial impact on reducing health care budgets. But what are the challenges that need to be addressed in its implementation in the clinic? This article poses some provocative questions and suggests some things that need to be considered.

“It is much more important to know what kind of patient has a disease than to know what kind of disease a patient has”  Caleb Parry, 18th century physician

The concept of personalized medicine has been around at least since the late 1990s. Then, ideas of rational drug design tailored to genomic profiles, or therapies developed to target individual genetic variation evolved from hopes and expectations raised by advances heralded directly or indirectly by the Human Genome Project, which undoubtedly has revolutionized our approach to biomedical research. But the aspirations that prevailed at the birth of the Genome Project anticipated more rapidly the delivery of safer, more effective medicines targeted more intelligently. This has just not been realized in the ensuing years. Our understanding has moved on from there and we now know that is it not only about genes; instead we now understand that emerging phenotypes in complex diseases are entirely dependent on the dynamics of interaction between genes and broader environmental, epigenetic influences.

But what does personalized medicine actually mean? It is a phrase that can be interpreted in a variety of ways, and anyway is not it the case that medical practice has always been “personalized”? Many physicians would certainly argue that is the case, but in general it is accepted that it is an approach to improve the delivery of novel therapies tailored to the needs of individual patients.

 So how does the current view of personalized medicine, increasingly championed as the route to improved health care, differ from current medical practice? Today, medicine is reactive, responding to the diagnosis of diseases late in their evolution, when they become symptomatic or at worst end stage. The move to a more predictive and preventive strategy, which lies at the heart of a personalized approach, embraces the concept of “right medicine, right patient, right time,” and is the driver for approaches that seek to integrate information from a variety of “-omic” sources, genetics, and lifestyle data. Some advocate that personal, targeted therapy will be driven by information derived from individual personal genome sequences (and companies have been established to deliver these); others believe that the approach is more likely to be one using current technologies to segment or stratify patient groups, using profiles that will identify those who are more likely to respond to treatments. In truth, both sequence/mutation-based rational therapies and stratified treatment regimes will emerge depending on the nature of the disease and what is practically feasible.

And what is practically feasible right now? Enormous advances have been made in recent years in developing a wide range of biomedical technologies, which, when applied in the research setting, have contributed to the description of diseases in increasingly fine molecular detail, and have helped to generate a tsunami of information. In contrast, clinical practice has largely remained reliant on the assessment of higher level phenotypic criteria and the use of algorithms to evaluate risk probabilities, rather than on the use of a deeper understanding of disease mechanisms gleaned from these technological advances. So, while many technologies are available for use, a gulf exists between exemplification of their potential in the research laboratory and their application in clinical practice, and this will influence what is considered by the jobbing physician to be practically feasible today. It may seem heretical to say it, but while many excellent research articles have been written in high impact journals, pushing the frontiers of scientific information and knowledge ever further forward, the intellectual advances alone are of little practical value unless they have an impact on medical practice or the crippling economics of health care. The challenge, therefore, is to find ways of translating the potential of cutting edge research excellence into clinical reality for the benefit of the patient and, ultimately, health care systems in general, in ways that can be incorporated relatively easily into routine medical practice by busy, pragmatic, conservative health care professionals, and without escalating costs.

And how is this to be achieved? It is interesting to note that many, if not all of the examples cited when discussing personalized medicine strategies, refer to studies in cancer. In the late 1990s, approvals of Gleevec and Herceptin raised hopes for a more rational targeted and personalized approach to therapy. More recently, innovative medicines targeting mutations in *bRaf* and anaplastic lymphoma kinase have been launched together with companion diagnostics. It is clear that the strong genetic drivers of disease progression in cancer provide an excellent platform to test and exemplify many of the technologies that might be applied routinely in personalization strategies for other diseases. But I would argue that these are very specialized cases because of the strong underlying genetic influences: if they are not themselves examples of diseases arising from major single gene defects, where mutation analysis has become part of routine clinical practice, neither are they the same as complex multifactorial diseases, where the interaction of a range of genes of small effect and environmental factors contribute to the emerging phenotype. While cancers are undoubtedly major causes of morbidity and mortality, and are diseases that raise many emotive debates on the relative costs and benefits of treatment in the context of quality of life, it is arguable that they are not the diseases that pose the greatest burden on health care budgets (Figure 1). The combined cost of inpatient and outpatient treatment, care, and loss of economic productivity in chronic, debilitating diseases such as circulatory and respiratory disease, dementia, diabetes and arthritis imposes a major drain on health care budgets. These complex multifactorial diseases are arguably more important to address in the context of personalization strategies if benefit to the greater proportion of populations and the consequent impact on reducing health care costs is to be achieved.

**Figure 1:**
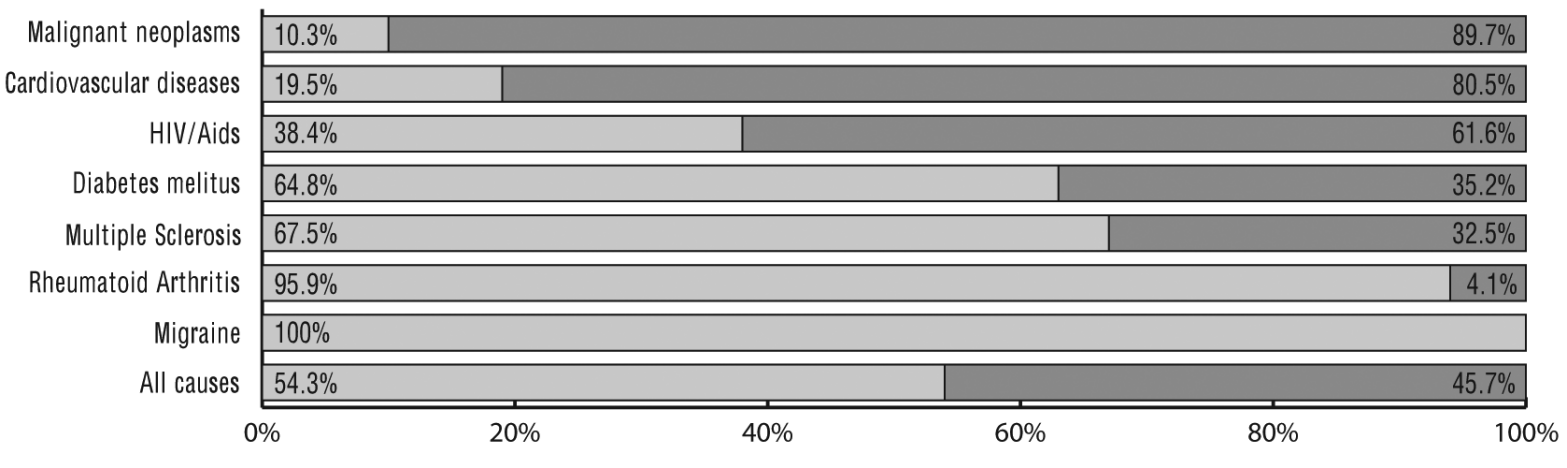
Disability (light gray) and mortality (dark gray) contribution to the total disease burden for selected diseases in Europe (WHO subregion EUR A). Source: Kobelt G, Kasteng F. Access to innovative treatments in rheumatoid arthritis in Europe, reproduced with permission from The Pharmaceutical Industry in Figures, EFPIA, 2010 edition (2).

This presents a significant problem for health care budgets in the 21st century. According to World Health Organization statistics (1), there is a disproportionately greater prevalence in the elderly of chronic, complex diseases that are debilitating and difficult to treat: cancer and heart diseases appear more frequently in 70-75-year olds than other age groups; 80% of circulatory diseases appear in the over 65s; and the risk of developing dementia rises steeply after the age of 60, with prevalence in men being greater due to increased longevity. The world population is increasing at a rate of about 80 million people per year and is estimated to reach around 8 billion by the year 2025. By that time, the proportion of those over the age of 65 is expected to represent 10% of the population, or some 800 million people. In Europe, the proportion of over 65s by that time will be closer to 25%. Thus, personalization strategies need to be focusing on making an impact here if they are to have genuine value to patient, health care systems, and national economies.

These diseases are areas of major focus for novel therapies, and have been for some time, but the success of bringing innovative medicines to market over the last couple of decades has been poor, despite the wealth of technical advancements experienced in medical sciences. Pharmaceutical industry figures (2) show that the full cost of bringing a new medicine to market has risen sharply since 1975, from some US $140 million to an estimated US $1300 million by 2006 (Figure 2). In contrast, the success of delivery of new medicines to market has declined significantly over the same period, despite our increased “knowledge” of disease mechanisms and pathways (Figure 3).

**Figure 2:**
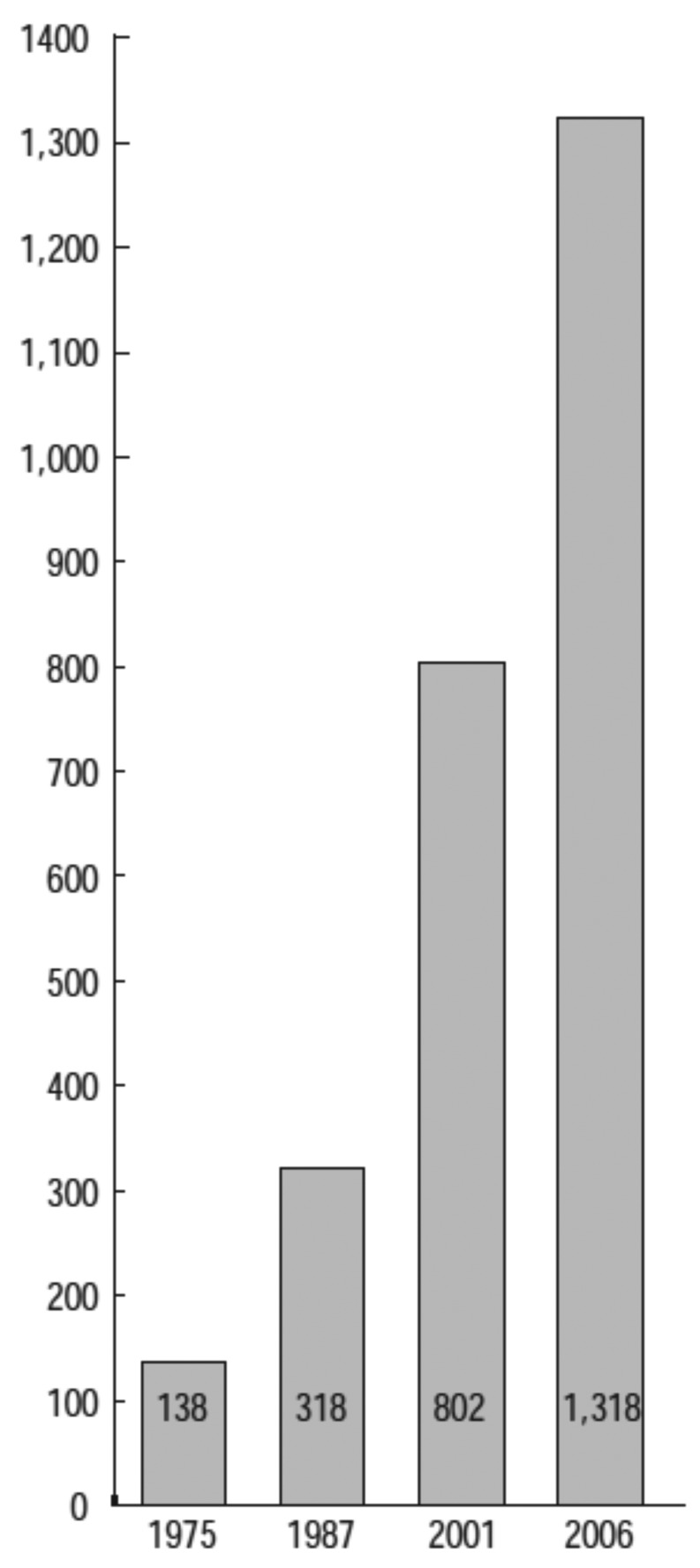
Estimated full cost of bringing a new chemical or biological entity to market (US $ million – year 2005 US $). Source: DiMasi JA, Grabowski HG. The cost of biopharmaceutical R/D: is biotech different, reproduced with permission from The Pharmaceutical Industry in Figures, EFPIA, 2010 edition (2).

**Figure 3:**
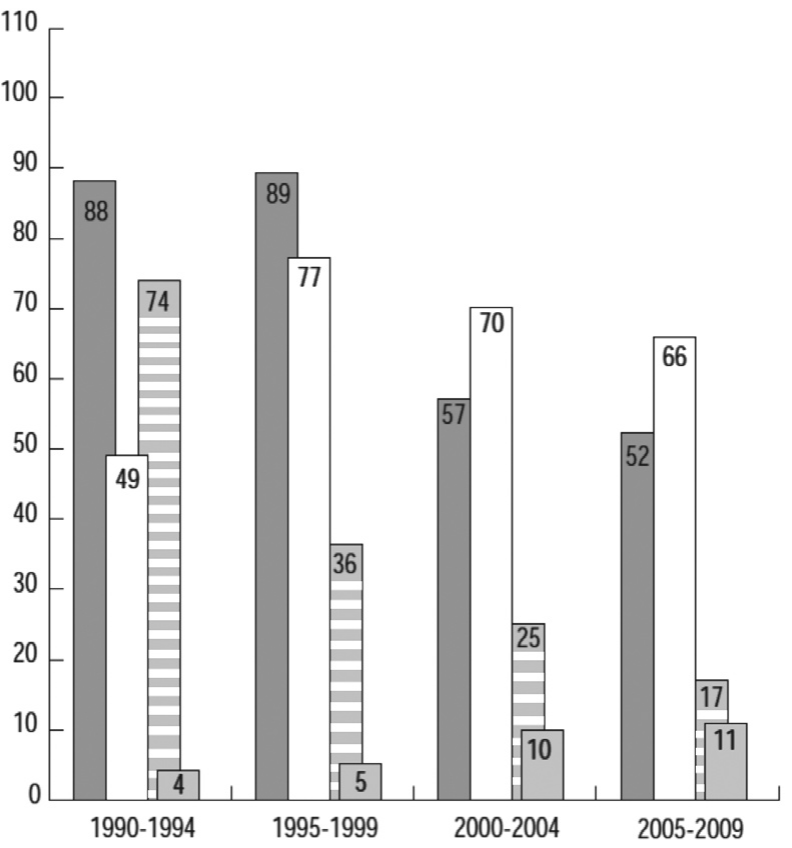
Number of new chemical or biological entities (1990-2009). Source: SCRIP-EFPIA calculations (according to nationality of mother company), reproduced with permission from The Pharmaceutical Industry in Figures, EFPIA, 2010 edition (2). Europe – dark gray; USA – white; Japan – horizontal lines; others – light gray.

Why are effective treatments for these complex diseases so hard to find? One major factor is, surprisingly, our relatively poor understanding of biology.

“Doctors are men who prescribe medicines, of which they know little, to cure diseases of which they know less in human beings of whom they know nothing” François-Marie Arouet (Voltaire, 1694-1778)

All of the technological advances over the last 20 years have done much to generate lots of information and data, but they have had little impact on improving our understanding of how individual components interact to enable function. Diseases are not the products of dysfunction in isolated individual entities or linear pathways; they arise from perturbations in complex dynamic networks that shift from patterns representing normal function to others that give rise to disease. Complex multifactorial diseases are not the result of a malfunction in a single target protein and are unlikely to be resolved by targeting a single gene product. However, the search for and development of new medicines focuses on reductionist, cell, and molecular screens that capitalize on the highly detailed information on targets that has been acquired as a result of the advances in molecular and cellular biology. The tendency has been to refine the optimization of novel compounds against isolated targets removed from their physiological networks, and then to “validate” and build associations to function in the human via a series of cellular and animal models, many of which are not faithful representations of the true physiological context in which the compound is ultimately intended to operate. There has also been an increasing problem with the toxic side effects of new medicines, although this has recently been improved significantly in the preclinical phase. Failure of medicines in development, especially in phase II or phase III is extremely costly and, according to recent statistics, is now largely related to a failure in efficacy (3). That is, the drug is not doing what it is supposed to do in the patient. So, even though we think we know a lot about the building blocks of biological systems, we actually know very little about how those building blocks operate in the physiological networks that underpin diseases, their progression, and the effect of any interventions that seek to improve outcome.

Putting it bluntly, how can such reductionist approaches possibly help us predict and test, let alone understand how a new medicine will work, when given to a patient in the target treatment group, which, in the case of the major illnesses that are being targeted by personalization strategies, is likely to be over 65, with a number of co-morbidities and already taking a number of other medications? So is it really that surprising to encounter failures in efficacy in the clinic? In our understandable enthusiasm to follow the path laid by the genome revolution, generating an evermore intricate map of human disease at the molecular level, we appear to have forgotten, or at least relegated the importance of an understanding of the physiological context.

The need now is undoubtedly to find ways to understand the dynamics of the complex interactions within the biological networks that underpin normal function, and the way in which these are perturbed in disease. We need to understand how targets operate not in isolation, but in physiological networks and, as importantly, within the context of the elderly who will likely present at clinic with a number of co-morbidities and taking a number of medications. This can be achieved through adopting integrative systems approaches that address the dynamics of disease networks, rather than analyzing static functions in isolation. The potential for these approaches to have an impact on 21st century medical practice is not in doubt: there are many publications clearly demonstrating this at the academic level, but what is lacking is sufficient evidence of its application in the real world and its ability to be reduced to practice. The challenge that faces us now, therefore, is how to evaluate and assess the added value that these advances in science bring to existing medical practice, and then to find ways of exploiting them routinely in the clinic. This task is complex and challenging, as it is dependent more than ever on the need to bring exploratory, blue skies science and busy, practicing physicians together to test the potential of, and troubleshoot, unproven technologies.

Those advocating a multidisciplinary approach, integrating the acquired molecular information by bringing together biology with mathematics, engineering, and physics have an increasingly strong voice in the biosciences. The use of mathematical models to tackle network complexity and to simulate and predict function is now seen as an inevitable evolution in biology. Indeed, industry analysts have gone so far as to say that it is an essential component of the changes that need to take place in the current pharmaceutical industry business model if it is to survive the challenges it faces (4).

And what about personalization? Historically the pharmaceutical industry blockbuster model has been to sell its products to as many patients as possible, so there has been a natural resistance to approaches that would segment their markets. But reality is now beginning to bite: if it can be shown that medicines can be targeted more accurately using new technologies, then it will not be long before payers begin to reimburse only in cases where companion diagnostics can demonstrate an increased potential for efficacy. The question here is, who will pay for the development of the diagnostic test and how will that get reimbursed?

With the increasing emphasis on personalization of therapies and the segmentation or stratification of treatment populations, the integrative systems approach described above is equally important in the context of biomarker and diagnostics development, helping to identify the needles in the haystack of potential options. The fundamental need, and the major challenge for biology and medicine now, is to improve our understanding of the connection between genetics and emerging phenotype through a better view of physiological systems behavior. This will require, not just the adoption of novel, cutting edge technologies, but a cultural change to embrace new business models and educational approaches, not just in the biosciences, but also in medicine.

“It’s not the strongest species that survive, nor the most intelligent, but the ones most responsive to change” Charles Darwin
